# A case of descending aortic rupture and aortoesophageal fistula secondary to fish bone–induced esophageal perforation

**DOI:** 10.1016/j.xjtc.2025.07.016

**Published:** 2025-07-28

**Authors:** Masashi Bungo, Ken-ichi Watanabe, Yoshio Teshima, Yuji Sakashita, Hisashi Uemura, Hiroe Tanaka, Mitsuhiro Yamamura, Taichi Sakaguchi

**Affiliations:** Department of Cardiovascular Surgery, Hyogo Medical University, Nishinomiya city, Hyogo, Japan

**Figure:**
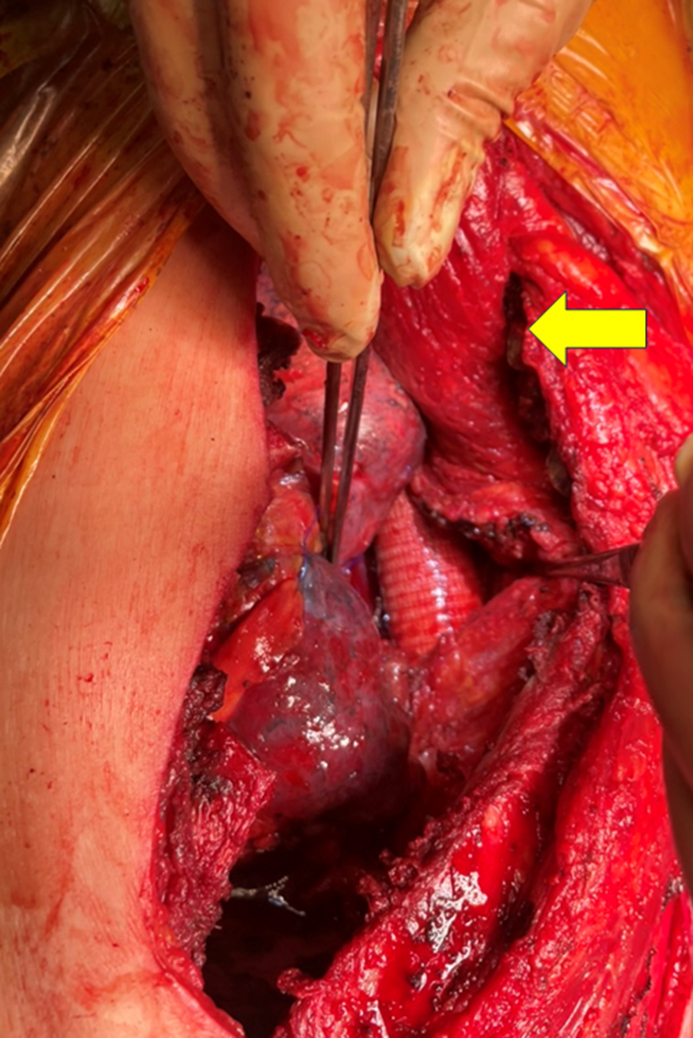
Pedicled latissimus dorsi flap lapping in aortic esophageal fistula surgery.

**Central Message**

The pedicled latissimus dorsi muscle flap procedure for an aortoesophageal fistula can be a viable alternative for infection control when omental coverage is infeasible.

An aortoesophageal fistula (AEF) is a rare but potentially fatal condition that often results from esophageal perforation. The standard treatment for AEF involves an esophagectomy with descending aortic replacement. The repair is typically covered with a flap of tissue taken from the omentum to prevent infection; however, omental flaps are contraindicated in some cases.

Here we present a rare case of AEF caused by fish bone–induced esophageal perforation that was treated successfully using a pedicled latissimus dorsi muscle flap. Written informed consent for publication was obtained from the patient; Institutional Review Board approval was not required for this case report.

## Case Description

**Figure 1:**
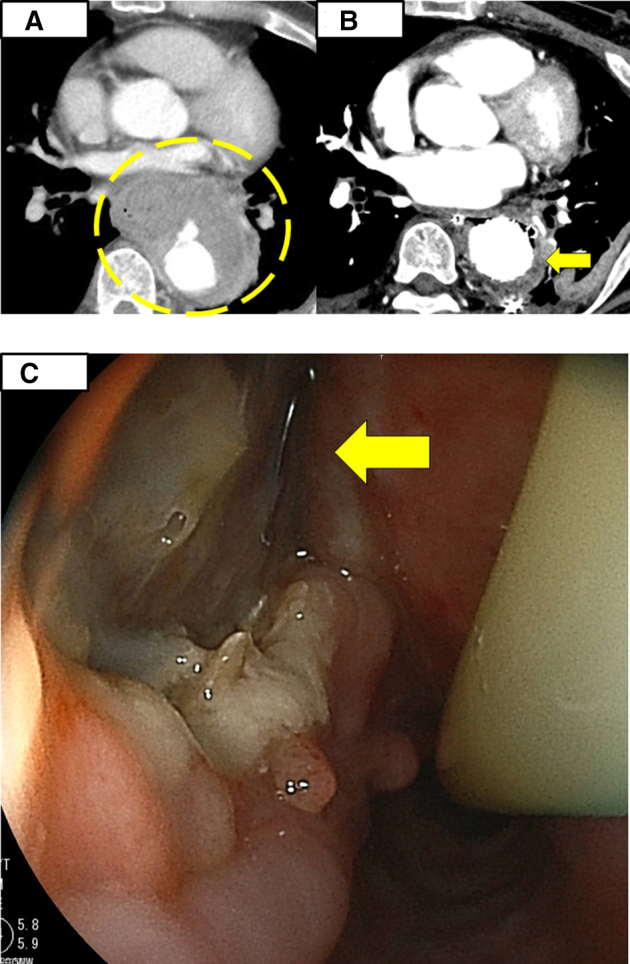
A, Computed tomography (CT) image showing a ruptured descending aorta (*yellow circle*). B, CT image showing free air surrounding the stent graft in the descending aorta (*yellow arrow*). C, Endoscopic image of the mid-esophagus revealing a fistula with the visible stent graft (*yellow arrow*).

A 65-year-old male patient presented with acute chest pain and was transferred to a local hospital. A contrast-enhanced computed tomography (CT) scan revealed a mediastinal abscess and a ruptured descending aorta (Figure 1, *A*). The patient underwent emergency thoracic endovascular aortic repair (TEVAR) (Gore TAG; 31 mm diameter, 10 cm long; WL Gore & Associates) and mediastinal drainage owing to his vital signs indicative of shock on hospital arrival. Subsequent upper gastrointestinal endoscopy performed 28 days after TEVAR revealed a mid-esophageal fistula with a visible stent graft, leading to the diagnosis of AEF. He was referred to our institution for further treatment.

The patient had no significant medical or family history, although he reported accidental ingestion of a fish bone approximately 1 month prior to admission. At our institution, a CT scan revealed the presence of free air surrounding the stent graft (Figure 1, *B*). This finding was subsequently confirmed by endoscopy, which revealed a persistent esophageal fistula (Figure 1, *C*). These findings indicated that the esophageal perforation was caused by a fish bone, which led to localized infection and subsequent rupture of the descending aorta and formation of an AEF.

Because the fistula did not close spontaneously, esophagectomy was performed 34 days after TEVAR. Bilateral intrathoracic manipulation during both esophagectomy and descending aortic replacement was anticipated to be highly invasive, and thus the decision was made to prioritize esophagectomy as the initial procedure. Esophagectomy was performed thoracoscopically. Following the procedure, a cervical esophagostomy was created, and a jejunostomy was established to facilitate postoperative nutritional management. Because *Staphylococcus aureus* was detected in the blood culture, administration of cefazolin (3000 mg/day) was continued postoperatively. Although a temporary improvement in inflammatory markers was observed after surgery, a recurrent elevation in the inflammatory response was noted on hospital day 15. On day 20 after admission, gallium scintigraphy revealed abnormal uptake around the stent graft, indicating a persistent infection.

Antibiotic treatment was continued, but there was little improvement in the inflammatory response. Consequently, descending aortic replacement with pedicled latissimus dorsi muscle flap coverage was performed on the day 41 day after admission.

**Figure 2:**
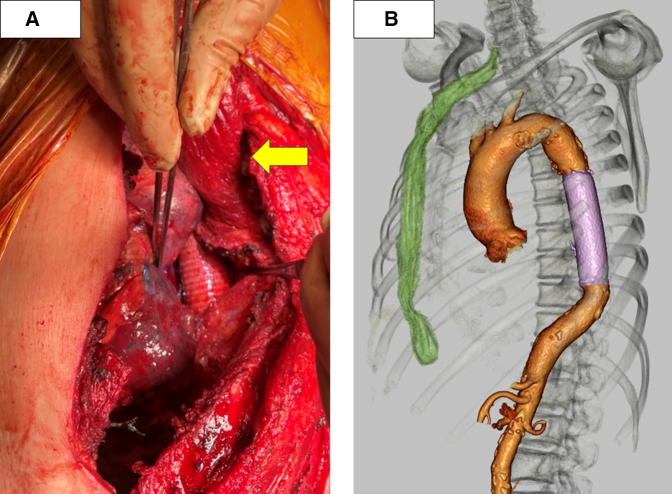
A, Intraoperative view after descending aortic graft replacement with pedicled latissimus dorsi muscle flap filling the thoracic cavity (*yellow arrow*).B, Postoperative esophageal reconstruction using a gastric conduit through a retrosternal route.

The patient was placed in the right lateral decubitus position, and a left posterolateral thoracotomy was performed via the third intercostal space. Ribs 4 to 7 were divided because of dense inflammatory adhesions. The presence of significant adhesions between the chest wall and the lung apex necessitated a wedge resection of the lung apex. The procedure involved replacement of the descending aorta with a prosthetic graft (J graft, 24 mm; Japan Lifeline Co, Ltd) performed under partial extracorporeal circulation. After graft implantation, a pedicled latissimus dorsi muscle flap was harvested and positioned within the thoracic cavity to wrap around the graft (Figure 2, *A*).

The patient's postoperative course was uneventful. On day 82 after admission, esophageal reconstruction was performed using a gastric conduit via the retrosternal route (Figure 2, *B*). He was discharged on day 106 after admission without complications. At the 2-month follow-up, there was no recurrence of infection or aortic complications.

## Discussion

AEF is a rare but catastrophic condition with a high mortality rate. Akashi and colleagues^1^ reported mortality rates of 46.8% at 6 months and 70.3% at 18 months among 47 surgically treated patients with an AEF, with infection the most common cause of death. Consequently, infection control is of utmost importance in AEF management. Particularly in the context of infection control, positron emission tomography scanning can be a complementary imaging modality for diagnosing peri-stent graft infection.^2^

The prevailing standard of care involves esophagectomy and prosthetic graft replacement, a combination that has yielded significantly superior outcomes compared with bleeding control alone or without esophagectomy.^3^ Graft coverage with an omental flap is widely recognized for its role in preventing infection by enhancing vascularity, supporting antibiotic penetration, and filling dead space^4^; however, in the present case, the use of an omental flap was contraindicated because of the need to preserve the omental space for planned gastric conduit reconstruction. Instead, a pedicled latissimus dorsi muscle flap was used to ensure adequate blood supply to the conduit.

Pedicled latissimus dorsi muscle flaps have a favorable prognosis in cases of prosthetic replacement of infected thoracic aortic aneurysms and stent graft infection after TEVAR.^5^ A pedicled latissimus dorsi muscle flap provides substantial tissue coverage extending to the diaphragm, thereby enabling secure and well-vascularized graft wrapping.

This case demonstrates that the use of a latissimus dorsi muscle flap is a viable alternative for infection control when omental coverage is not feasible.

## Conclusions

The use of a pedicled latissimus dorsi muscle flap may be a valuable surgical option for infection control in AEF cases in which omental flap coverage is not possible.

## Conflict of Interest Statement

The authors reported no conflicts of interest.

The *Journal* policy requires editors and reviewers to disclose conflicts of interest and to decline handling or reviewing manuscripts for which they may have a conflict of interest. The editors and reviewers of this article have no conflicts of interest.
